# Modeling and Analysis of the Capillary Force for Interactions of Different Tip/Substrate in AFM Based on the Energy Method

**DOI:** 10.1021/acsmeasuresciau.3c00001

**Published:** 2023-03-07

**Authors:** Amir Farokh Payam

**Affiliations:** Nanotechnology and Integrated Bioengineering Centre (NIBEC), School of Engineering, Ulster University, Belfast BT15 1AP, United Kingdom

**Keywords:** Capillary Force, Tip Shape, Humidity, Contact Angles, Meniscus Bridge

## Abstract

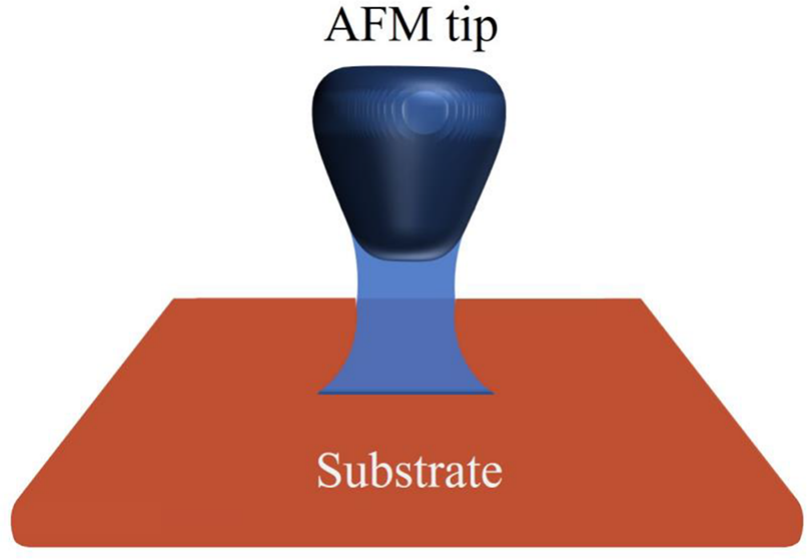

This paper presents a simple and robust model to describe the wet adhesion of the AFM tip and substrate joined by a liquid bridge. The effects of contact angles, wetting circle radius, the volume of a liquid bridge, the gap between the AFM tip and substrate, environmental humidity, and tip geometry on the capillary force are studied. To model capillary forces, while a circular approximation for the meniscus of the bridge is assumed, the combination of the capillary adhesion due to the pressure difference across the free surface and the vertical component of the surface tension forces acting tangentially to the interface along the contact line is utilized. Finally, the validity of the proposed theoretical model is verified using numerical analysis and available experimental measurements. The results of this study can provide a basis to model the hydrophobic and hydrophilic tip/surfaces and study their effect on adhesion force between the AFM tip and the substrate.

## 1 Introduction

The study of capillary force has a long-time history and is considered a multidisciplinary field of research. Particle coalescence/agglomeration resulting from liquid bridge adhesion plays a significant role in various vital areas such as the pharmaceutical and food industry,^1−3^ soft matter and cell biology,^4,5^ controlling the fluid flow,^6^ and solid–liquid interfaces.^7^

Capillary force is a meniscus force due to condensation typically observed in a wet medium. To model and simulate the capillary force, the nonlinearity of the capillary equation combined with the variety of substrate geometries leads to a large number of solutions.^8−15^ Generally, the modeling approaches for capillary force can be classified into two main categories. The first one is based on the integration of the liquid bridge profile with the Laplace–Young equation,^9^ while the second approach relies on the total energy of the liquid bridge.^16,17^ Due to the simplicity of calculation, the energy approach gets more attention. The usual way to model the capillary force in this approach is a simplification of hydrostatic pressure across the liquid–air interface and subsequently exploits a gorge,^18^ circular,^3^ parabolic,^19^ and toroidal^20^ approximation. However, the main limitations of these methods are assumptions or approximations of the bridge profile including distance, volume, and embracing angle.

To measure capillary force, an atomic force microscope (AFM) offers significant advantages and received considerable attention,^3^,^10^,^11^,^16^.^21−27^ Using different tip shapes, flexible measurements of materials with various properties, and the possibility of particles glued to the tip apex are the main factors that make AFM a versatile tool for capillary force measurements.^26^ To analyze the AFM experimental observables and quantify the measured capillary force, modeling the wet adhesion between different object shapes and particles is required. The basis of most presented models is the energy method or a direct force calculation from the meniscus geometry obtained by the solution of the so-called Laplace equation,^28^ consisting of several approximations. For example, Farshchi Tabrizi et al.^24^ and Jang et al.^25^ assumed equal contact angles for tip/liquid and liquid/plane interfaces in conjunction with a simple geometry for the AFM tip. In another approach, the capillary equation is solved by a numerical algorithm without providing any analytical and mathematical explanation.^23,26^

In this paper, to model the capillary force, I consider either equal or nonequal contact angles for two solid/liquid interfaces, i.e., AFM tip/liquid and liquid/substrate interfaces. Then based on the integration of the capillary adhesion resulting from pressure difference across the free surface and the surface tension force, the different AFM tips in the wet interaction by the substrate are modeled and analyzed. Hence, in section 2, the formation of the liquid bridge and the energy method is discussed. Then in sections 3 and 4, the capillary force for the two main AFM tip shapes, sphere and two-sphere shape, is calculated.

In section 5, I investigate the effects of the tip/sample distance, humidity, contact angles, and tip geometry on the capillary force for both symmetric and asymmetric cases, and the results of the numerical study are presented. A comparison between the proposed analytical method with the experimental measurement is given in section 6. Finally, in section 7, a conclusion is provided.

### 2 Capillary Force Formation

**Figure 1:**
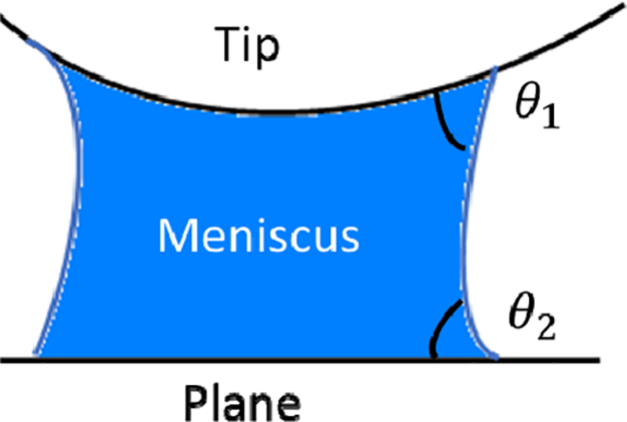
Schematic of the capillary force formation.

Figure 1 presents the geometrical schematic of the capillary force problem. Due to the humidity condensation, a tip and plane are linked through a liquid (usually water) meniscus. θ_1_ and θ_2_ are the contact angles for the two-phase interfaces (tip/liquid and liquid/plane).

When the radius of curvature of the nanocontact is below a certain critical radius a meniscus will be formed. This critical radius is defined approximately by the size of the Kelvin radius:
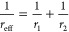
1

Where *r*_1_ and *r*_2_ are the principal radii of the curvature while the former is negative and the latter is positive.^17^ The Kelvin radius is connected with the partial pressure *P*_s_ (saturation vapor pressure) by^28^
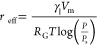
2

With the assumption of the liquid is formed by water vapor, γ_l_ = 0.072(*N*/*m*) is the surface tension, *R*_G_ = 8.268 (J/Kmol) is the gas constant, *T* = 298(*K*) is the temperature, *V*_m_ = 18 × 10^–6^ (m^3^/mol) is the mol volume and *P*/*P*_s_ is the relative vapor pressure (relative humidity). In general, the meniscus force is the sum of a capillary pressure force caused by the reduced pressure inside the meniscus and the surface tension component, which is a direct result of the surface tension of the liquid.

To describe the shape of the liquid meniscus, the circular approximation,^12^,^25^^29^ is assumed. To consider the effect of adhesion force due to the pressure difference between outside and inside the liquid bridge, the energy method is utilized. Subsequently, the capillary force is modeled by the integration of the energy method^30^ and the vertical surface tension force.

The capillary pressure energy of a liquid bridge is the sum of two contributions: the liquid–solid (*γ*_sl_*A*_sl_), and the solid–vapor (*γ*_sv_*A*_sv_) interfacial energies, where γ and *A* denote the respective surface tensions and surface areas.

The capillary pressure energy *W*_p_ is given by
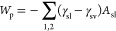
3

Note that following the methods proposed by previous investigations,^12,24,27^ the attractive force is considered to be positive.

Using the Young–Dupré equation, interfacial energies can be substituted by contact angle and surface tension:
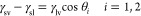
4

So the energy equation is converted to
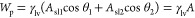
5

And the capillary force is given by
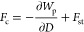
6

Where *F*_st_ is the surface tension force.

## 3 Capillary Force between a Sphere Tip and a Solid Plane

**Figure 2:**
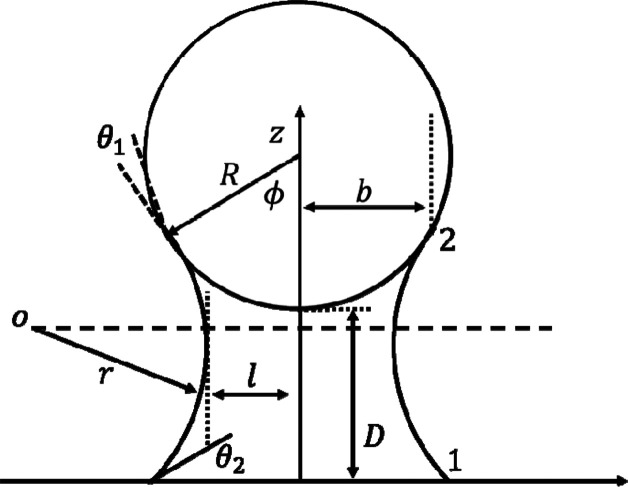
Model for a sphere tip in wet adhesion with a substrate.

The schematic geometry of a sphere tip and substrate is illustrated in Figure 2.

In this figure, φ is the filling angle, and *R* is the radius of the circle of the liquid bridge wetting the sphere.

The volume of the liquid bridge *V*_l_ is obtained from
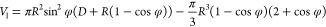
7

On the other hand, the relevant areas of eq 5 are given by
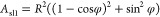
8
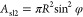
9
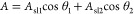
10

To calculate the capillary force, we need to take the derivative from the energy with respect to the AFM tip and substrate distance. So:
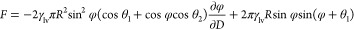
11

Note that ∂φ/∂*D* is calculated based on the following condition:
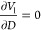
12

So, ∂φ/∂*D* is obtained as
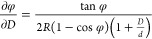
13

Where
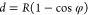
14

Substituting ∂φ/∂*D* in (11), the capillary force is calculated by
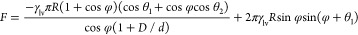
15

It is worth mentioning that the presented model is limited by the value of the filling angle to be in the range that the calculated relative humidity does not increase by more than 1, which has no physical meaning.

## 4 Capillary Force between a Two-Sphere Tip and a Solid Plane

**Figure 3:**
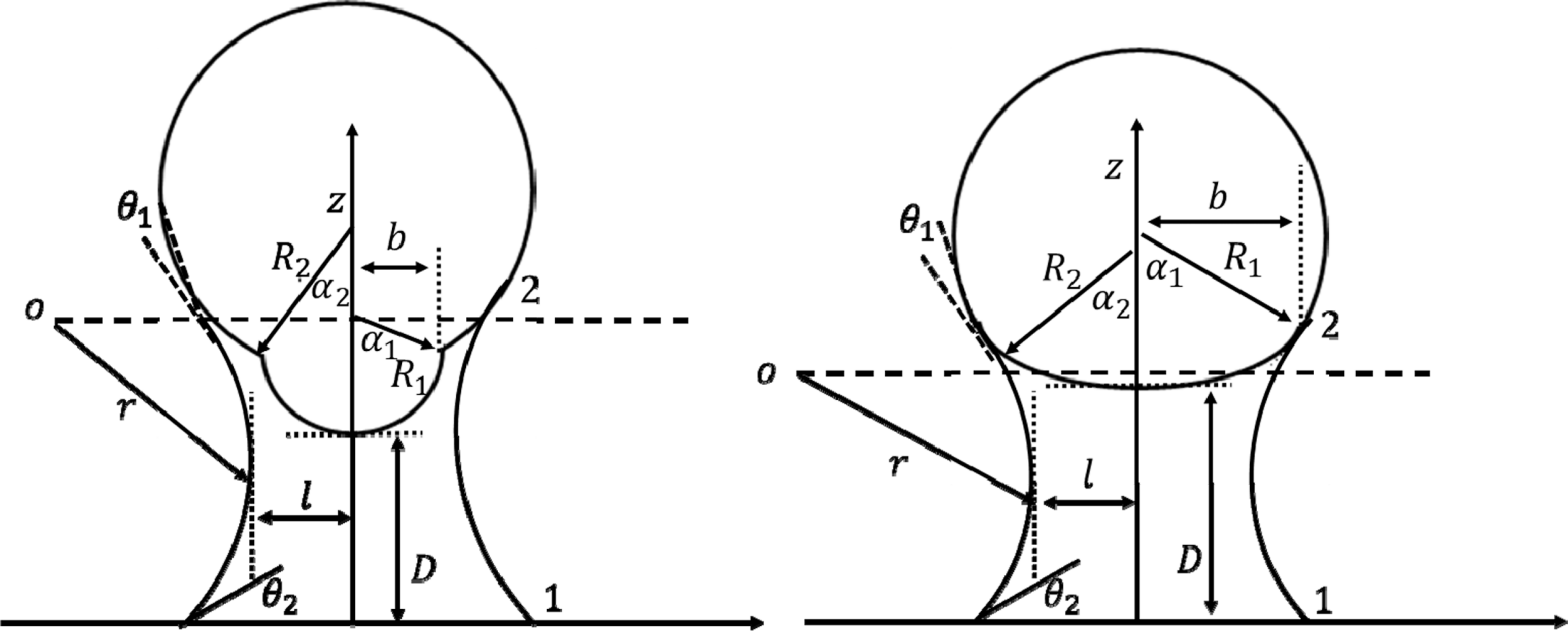
Schematic of a capillary interaction between a two-sphere tip and the plane.

The schematic of the analysis model for a sphere tip and the flat substrate is shown in Figure 3. In this figure, the radius of spheres is depicted by *R*_1_, *R*_2_; α_1_ is the maximum of the half opening angle of the part of sphere 1, and α_2_ is the minimum half of the opening angle of sphere 2; *b* is the circle radius of the water meniscus wetting the sphere tip; and *D* is the distance between tip and substrate.

For *b* < *R*_1_ sin α_1_ = *R*_2_ sin α_2_, the liquid bridge is formed between the first spherical end of the tip and substrate (*R*_1_). For this case, the procedure is identical to the case of sphere tip in section 3.

For *b* ≥ *R*_1_ sin α_1_ = *R*_2_ sin α_2_ the liquid bridge is formed on the sphere 2 boundary. In this case, the volume becomes
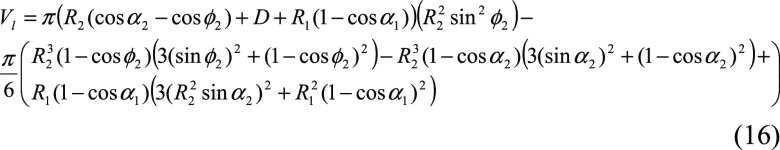
16

The relevant areas for the eq 5 are given by
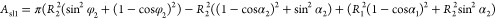
17
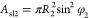
18

To calculate the capillary force, we need to take the derivative of the energy with respect to the distance:
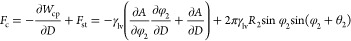
19∂φ_2_/∂*D* is calculated based on the following condition:
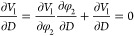
20

Substituting ∂φ_2_/∂*D* in (19), the capillary force is calculated.

## 5 NUMERICAL RESULTS AND DISCUSSION

### 5.1 Spherical Tip and Substrate

**Figure 4:**
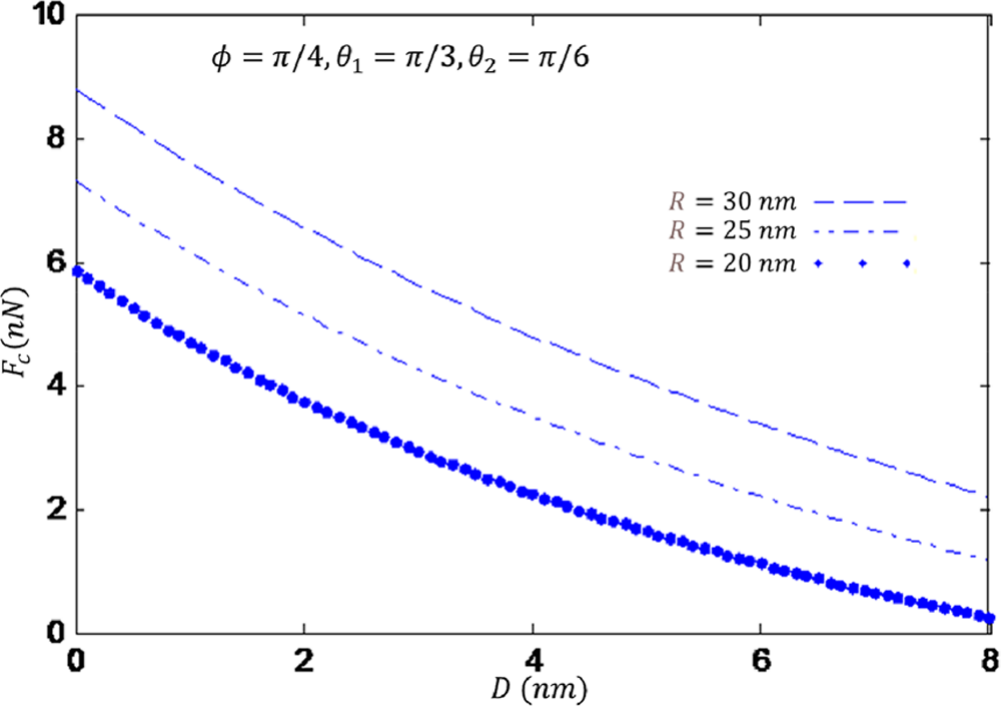
Capillary force as a function of the distance between tip and substrate.

Figure 4 illustrates the relationship between the AFM tip, the distance between the tip and substrate, and capillary force. By increasing the distance, the capillary force is reduced. Also, decreasing the sphere tip radius leads to a reduction in capillary force.

**Figure 5:**
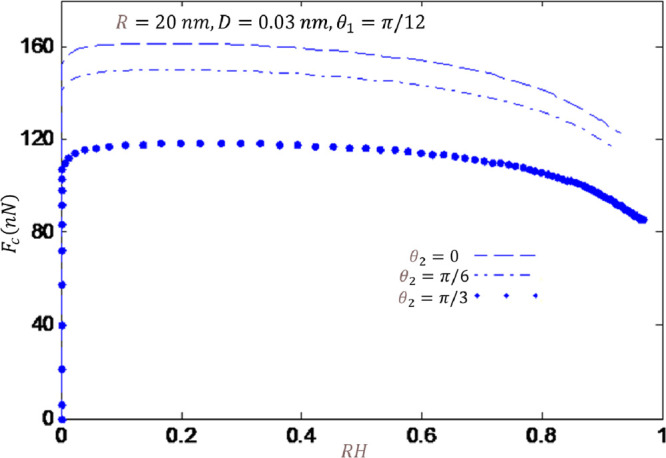
Capillary force versus humidity for different contact angles.

Figure 5 shows the dependency of capillary force on the humidity. In this case, I consider the effects of contact angles. So, by keeping the fixed distance between the tip and substrate, the contact angles are changed. From the results, I conclude that as the contact angle between the substrate and liquid increases, the capillary force decreases. So, hydrophobicity of the surface leads to a reduction of capillary forces.

Furthermore, for all three different contact angles, the trend of capillary force behavior with changes in relative humidity is the same. As the results show, increasing the relative humidity first increases the capillary force, then at higher relative humidity, the capillary force decreases.

### 5.2 Two-Sphere Tip and Substrate

**Figure 6:**
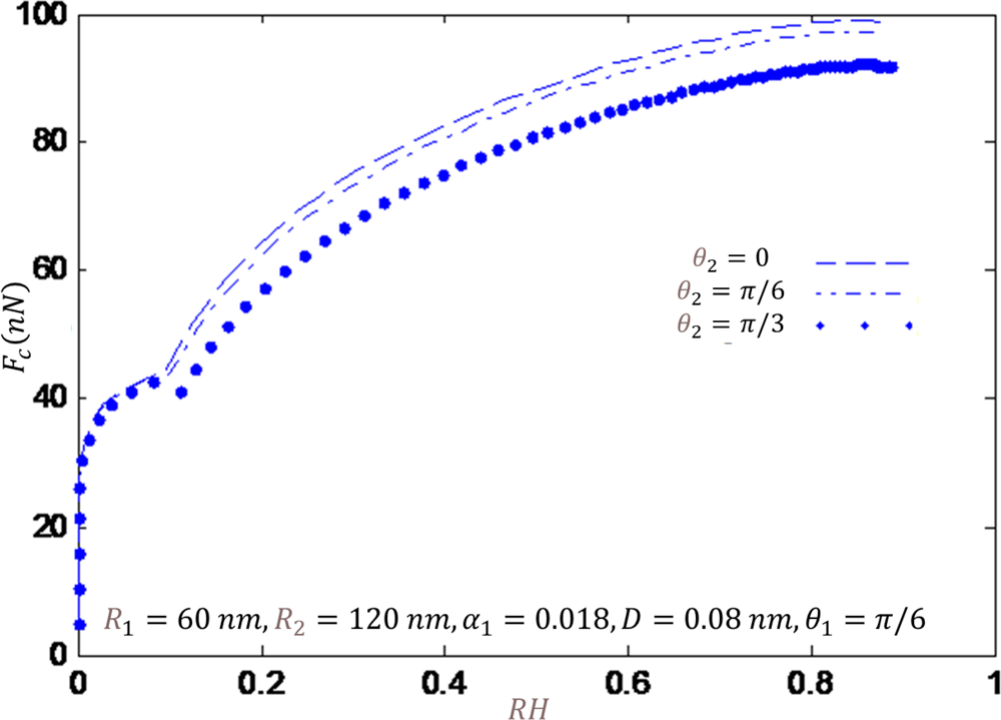
Capillary force versus humidity for a sharp tip and different contact angles.

**Figure 7:**
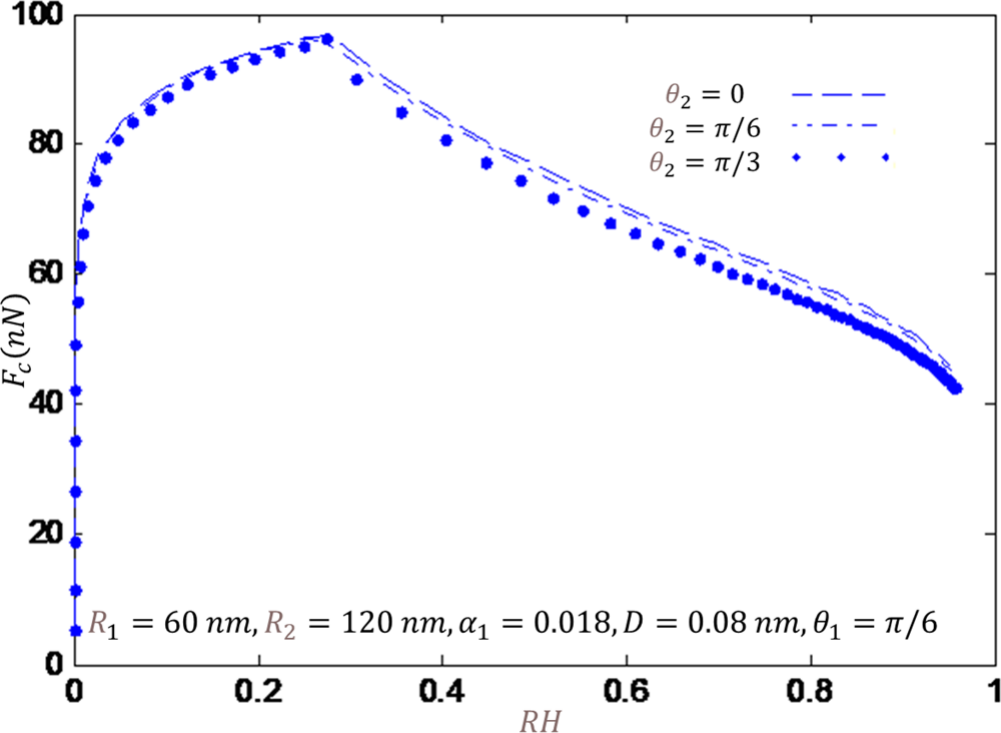
Capillary force versus humidity for a blunt tip and several contact angles.

In this numerical analysis, I consider two cases. In the first case, I select a sharp tip. As Figure 6 shows, by increasing the humidity, the capillary force increases. Also, the results demonstrate the transition region between the two radii. The results of the second case are reported in Figure 7 as I consider the blunt tip. Here, I use the two-sphere model to calculate the capillary force.

**Figure 8:**
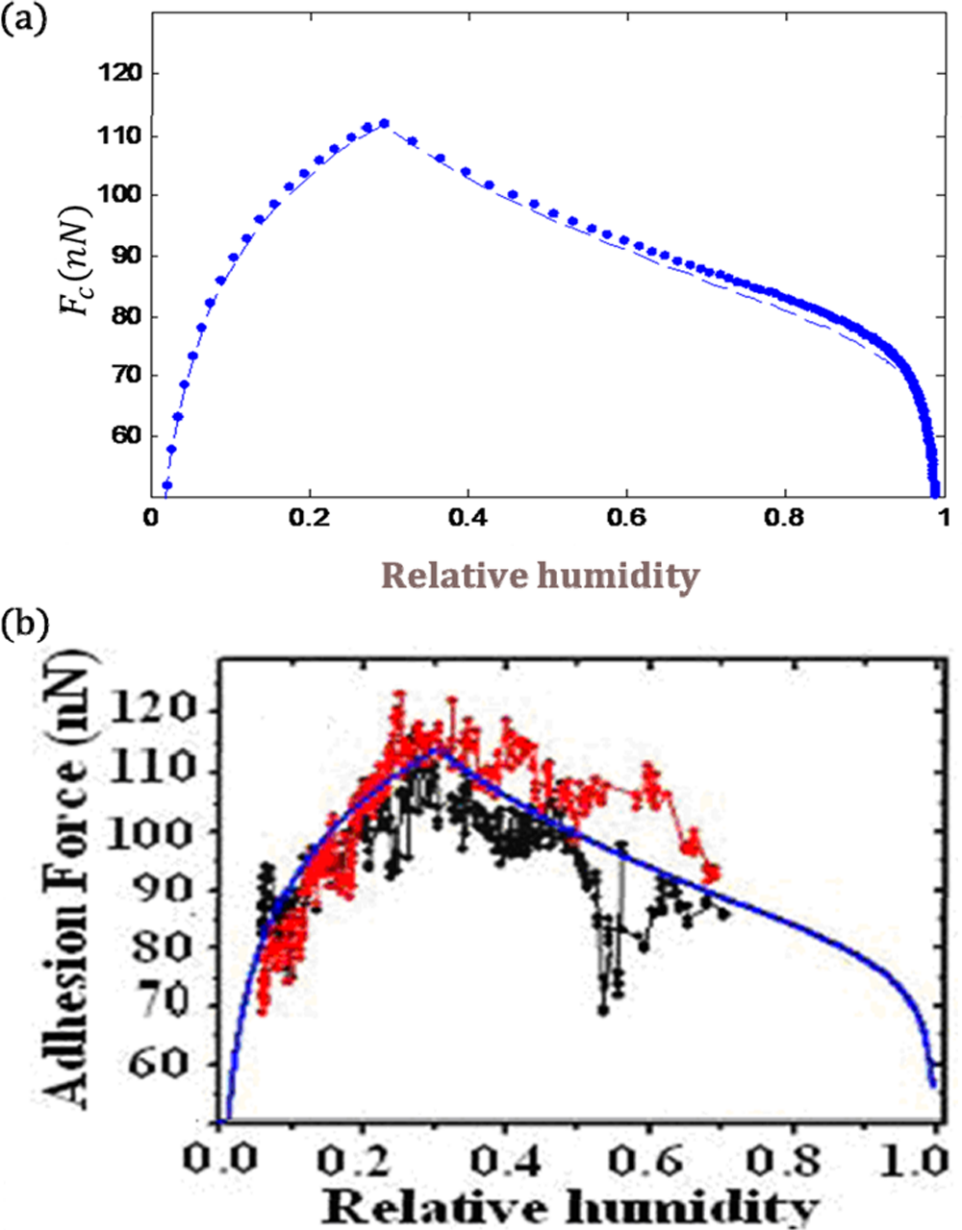
(a) The simulation of the experimental measurement in refs (24,31) with the presented model and comparison with the (b) experimental measurements. Reprinted (adapted) with permission from ref (24) {*Langmuir***2006**, *22*, (5), 2171–2184}. Copyright {2006} American Chemical Society.

From these results, one may infer when the wet circle boundary is located on the first sphere, the capillary force increases as the humidity increases. In contrast, in the transition region between two spheres, the capillary force decreases by increasing the humidity. From these results, it can be inferred that in the case of the two-sphere model, the effect of relative humidity on capillary force is dependent on the tip shape. For the sharp tip, increasing relative humidity leads to an increase of capillary force. While for the blunt tip, the same as the sphere tip, the capillary force decreases by increasing the relative humidity. Furthermore, according to the physical understanding, a decrease in the contact angles and the gap between the tip and substrate increases the capillary force. This result can explain the observed behavior of the hydrophilic surface in increasing the capillary force.

## 6 COMPARISON WITH EXPERIMENTAL RESULTS

In this section, to examine the validity of the presented approach, I regenerate the experimental curves of adhesion force versus humidity. As can be seen, high proximity exists between the proposed model and the experimental measurements. It is worthwhile to mention that Figure 8 (—) is taken from^31^ and regenerated here for comparison purposes. The simulation parameters are selected from the experimental measurements of ref.^31^ As shown in Figure 3.11-C of ref,^31^ the tip shape can be modeled as a two-sphere shape (Figure 3).

## 7 Conclusion

In this paper by considering two main AFM tip shapes and a flat substrate, based on the combination of energy method to calculate adhesion forces due to the Laplace pressure and surface tension force, the capillary adhesion force is calculated. The basis of the models is a circular interface in a radial cross-section. In addition to simplicity and robustness, the main advantage of the proposed method is the analytical solution of capillary force equations while avoiding several approximations considered in the previous analytical approaches. Moreover, as an advantage of the proposed approach, we can model the symmetric and asymmetric cases for the meniscus geometry as representative of hydrophilic and hydrophobic surfaces. Finally, by the numerical analysis and comparison with the available measurement data, derived models are examined, and I verify the accuracy and precision of the calculations.
